# A Simplified Method for Extracting the Movement Trajectories of Small Aquatic Animals

**DOI:** 10.3390/mps8040067

**Published:** 2025-06-20

**Authors:** Xin Liu, Huanan Gao, Aimin Hao, Yasushi Iseri

**Affiliations:** 1 Guangxi Key Laboratory of Aquatic Biotechnology and Modern Ecological Aquaculture, Guangxi Academy of Marine Sciences, Guangxi Academy of Sciences, Nanning 530007, China; 2 School of Environmental Science, The University of Shiga Prefecture, Hikone 5228533, Japan; 3 Center for Ecological Research, Kyoto University, Otsu 5202113, Japan; 4 Environmental Simulation and Pollution Control State Key Joint Laboratory, State Environmental Protection Key Laboratory of Microorganism Application and Risk Control (SMARC), School of Environment, Tsinghua University, Beijing 100084, China; gaohn@mail.tsinghua.edu.cn; 5 College of Life and Environmental Science, Wenzhou University, Wenzhou 325035, China; iseri@wzu.edu.cn; 6 Sanyang Wetland Ecological Environment Research Institute, Wenzhou University, Wenzhou 325000, China

**Keywords:** freshwater copepod, movement tracking, swimming behavior, two-dimensional observation system, zooplankton

## Abstract

Understanding the motion behaviors of animals is crucial for unraveling the mechanisms underlying ethology across various domains, such as movement patterns, food detection, and defense strategies. In this study, we devised a simplified method enabling the movement of small animals to be tracked conveniently using high-resolution smartphone videos and freely available tracking software. Employing a laboratory video setup, we traced the swimming trajectory of the small copepod zooplankton *Eodiaptomus japonicus*, which has a body size of approximately 1 mm. From the tracked position data, we analyzed key motion parameters, including swimming distance, speed, and jump frequency. The results of our video analysis showed that adult female *E. japonicus* exhibited an average swimming speed of 9.8 mm s^−1^, displaying a predominant cruising pattern with speeds of around 5.0 mm s^−1^, punctuated by sporadic jumps, showcasing maximum instantaneous speeds reaching a remarkable 190.1 mm s^−1^. Our successful tracking of the high-speed swimming copepod not only sheds light on its locomotion dynamics but also underscores the potential to refine this method to study the motion trajectories of diverse animal species.

## 1. Introduction

Movement is an important life history trait in organismal ecology. Individual movement decisions and capacities affect habitat-dependent space-use and foraging strategies, as well as dispersal and migration [1,2,3]. Motion tracking can be an efficient way to monitor animals’ movement [4,5]. Video analysis is a convenient approach that enables motion tracking and consequently helps in obtaining behavioral information and capturing dynamic processes. It also has the potential to describe the interactions between individuals [4,6,7]. The movement behavior of animals is a critical aspect of biology and ecology, with profound implications for understanding behaviors such as mating and food seeking [8,9] and the responses to environmental changes and population dynamics [10,11,12]. These parameters can offer insights into the ecological roles and physiological adaptations of a vast array of species [6,10,13].

Different species have evolved a variety of moving techniques and speeds suited to their ecological niches [14]. Technological advancements have revolutionized animal tracking, enabling more precise and comprehensive studies [1,15]. For large animals, modern tracking devices, for example, Global Positioning Systems (GPSs) or the Advanced Research and Global Observation Satellite (ARGOS), make it possible to gather data at increasingly fine spatiotemporal resolutions, thereby providing the data necessary to address comprehensive questions about how individuals perceive, utilize, and/or respond to environmental changes [16,17]. To quantify the abundance and migration of small aquatic animals such as zooplankton, who have a body size spanning only several millimeters, tools such as ZooScan and Video Plankton Recorder are being developed [18,19]. Zooplankton movement tracking is mostly performed in the laboratory. Previous studies have used a highly technological video setup with a two- or three-dimensional observation system to carry out zooplankton movement tracking experiments [4,7,20], and swimming trajectory data are mostly extracted using LabTrack software (BIORAS, Ebeltoft, Denmark) [4,10,21].

*Eodiaptomus japonicus* is an endemic calanoid copepod that dominates the zooplankton community in Lake Biwa, the largest lake in Japan, throughout the year [22,23]. This omnivorous copepod selectively feeds on nano- to micro-sized particulate organic matter, including phytoplankton and detritus, as well as microzooplankton, such as rotifers and protists [23]. A previous study using a three-dimensional observation system clarified its mate-seeking behavior, demonstrating that this species employs hydromechanical signals to remotely detect prospective mates [20]. In contrast, a two-dimensional observation system has been designed to investigate the resource acquisition mechanisms and locomotion dynamics in small aquatic animals [24]. These multidimensional observation systems not only deepen our understanding of species-specific behaviors but also have broader applications in fields such as biomechanics and ecology. Given the prohibitive costs associated with the existing video setups and motion trajectory software, this study aimed to develop an affordable system for studying the movement behavior and characteristics of small, fast-moving animals. It provides insights into the potential benefits of this tracking method for ecological research.

## 2. Materials and Methods

### 2.1. The Sample Collection

Adult female calanoid copepods (*E. japonicus*) were sorted from zooplankton samples collected using vertical plankton net hauls (diameter, 45 cm; mesh size, 200 μm) from 30 m to the surface at a sampling site in the north basin of Lake Biwa (70 m deep, 35°18′32.6″ N, 136°8′38.9″ E) on 13 May 2021. The copepods were maintained in the laboratory using a procedure described in a previous study [25,26] until swimming tracking. Because female *E. japonicus* copepods are easier to handle due to their slightly larger body size compared to that of their male counterparts [25,26], we selected female copepods for the swimming tracking experiment. Our previous study showed no significant difference in swimming behavior between genders in this copepod species [20]. The movement patterns in small copepod species are independent of vessel size and density to a limited extent, at least up to ~160 ind. L^−1^ [27]. Prior to the swimming tracking experiment, one active and healthy adult female specimen of *E. japonicus* (with an approximately 1 mm prosome length) [25] was placed into the well (diameter: 35 mm) of a six-well culture plate (IWAKI, 1810-006-MYP) filled with 10 mL of filtered lake water (an equivalent individual density of 100 ind. L^−1^), a volume sufficient to assess its swimming behavior [28], and acclimated in a 20 °C incubator (Sanyo, MLR350, Tokyo, Japan) for at least one hour to avoid physical shock.

### 2.2. Video Techniques and Quantification and Analysis of Swimming Behavior

The two-dimensional video setup involved a smartphone (HUAWEI P30, Shenzhen, China). This smartphone features a built-in Leica camera (~40-million pixels) with excellent imaging and video recording capabilities. Phototactic or photophobic responses in copepods may occur during light flashes or directed light beams [29,30]. In this video setup, continuous uniform illumination was provided using light-emitting diodes in the visible-light wavelength range, approximately 400–700 nm [31], positioned beneath the six-well culture plate with a translucent frosted plastic cover to avoid positive and negative phototactic responses in the studied copepods (Figure S1). The camera overlooked one well of the culture plate, and the setup was adjusted to ensure that the copepods were adequately resolved and in focus (Figure S1). After one hour of acclimation, when the setup was complete, quantification of the pathways of individual swimming was conducted, and the copepods swam freely. Two-dimensional projections of the swimming tracks were manually digitized using Fiji software (Version 2.1.0/1.53c, https://imagej.net/Fiji, 6 June 2025) using a movement tracking package (Manual Tracking). To ensure accuracy and minimize the bias resulting from optical distortion, we tracked the trajectory of the copepod’s central point, which served as a reliable dataset for analyzing its swimming behaviors. The instantaneous swimming speed (at the smaller scale of 1/30 s) was estimated as follows. At a generic time step *t*, the instantaneous swimming speed *V*_t_ (mm s^−1^) between two points in the two-dimensional space was computed from the *x* and *y* coordinates following a modified equation [4]:

(1) $$V_{t}=\sqrt{{{(x}_{t+1}-x_{t})}^{2}+{{(y}_{t+1}-y_{t})}^{2}}\times \alpha \times p$$

where (*x*_t_, *y*_t_) and (*x*_t+1_, *y*_t+1_) are the positions of the copepod at time *t* and *t* + 1, respectively; α is the distance of 1 pixel in frame (α = 32.407 μm px^−1^, calibrated with the standard scale); and *p* is the frame refresh rate of the camera (*p* = 30 frames s^−1^). The frequency of swimming speeds was also provided, and swimming speeds were determined as the average of all instantaneous swimming speeds calculated across the individual track. The data analysis was performed using MATLAB software (Version R2021b) [32].

## 3. Results

**Figure 1:**
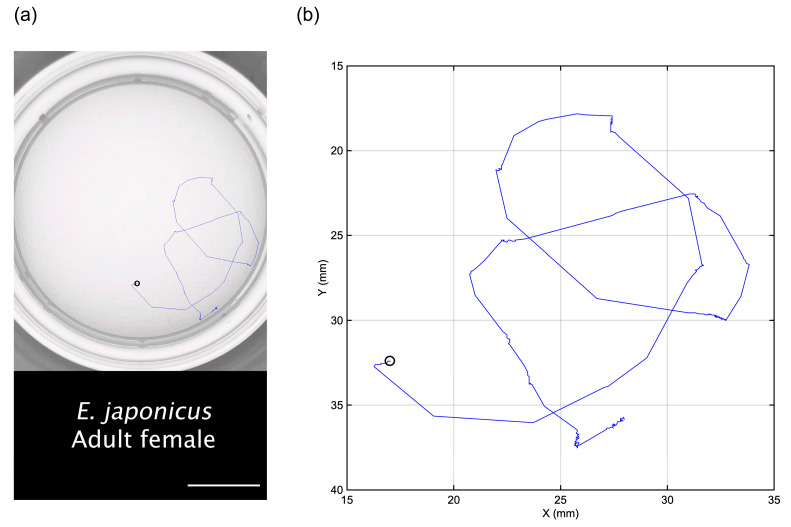
The top view of a ten-second swimming trajectory (blue line) of an adult female specimen of *Eodiaptomus japonicus* (**a**). The scale bar represents 10 mm. A plot generated using the position dataset extracted using the Fiji software with the Manual Tracking package is also shown (**b**). The black circle indicates the starting point.

**Figure 2:**
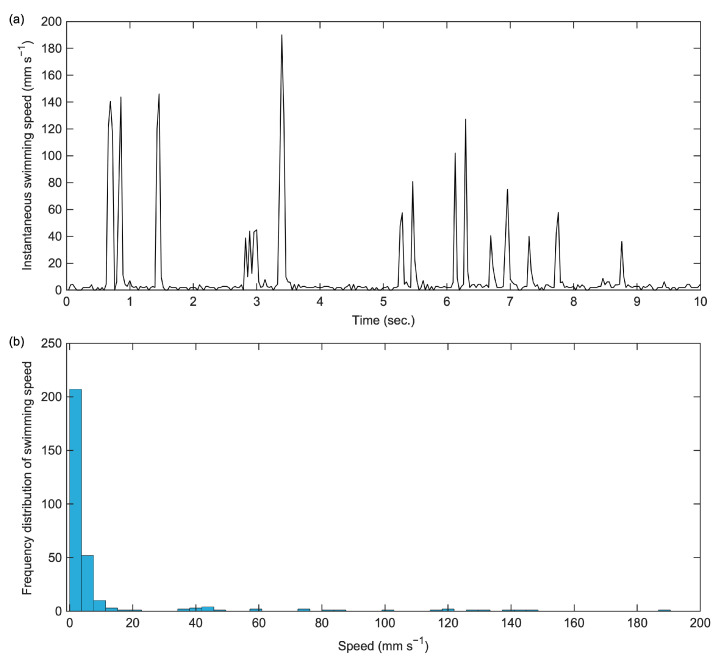
The instantaneous swimming speed (**a**) and frequency distribution of swimming speed (**b**) of the adult female *Eodiaptomus japonicus* specimen over a ten-second trajectory.

An original video of the swimming behavior of an adult female *E. japonicus* is presented in Video S1. In this video, a single swimming individual is clearly visible, proving that copepod swimming can be recorded effectively using the aforementioned smartphone camera. The tracking trajectory of this individual, extracted using tracking tools, is illustrated in both Figure 1 and Video S2. The tracking trajectory remains distinguishable even when the individual swims along the edge of the well, demonstrating the effectiveness of the tracking method. In a ten-second tracking video, the total movement distance of the copepod was measured at 98.5 mm (Figure 2). The analysis revealed two distinct swimming patterns: a cruising pattern and a jump pattern. The average swimming speed was 9.8 mm s^−1^, though the most frequently observed speed was below 5.0 mm s^−1^ (Figure 2). Over the tracking period, we identified 16 spontaneous jumps (Figure 2a), characterized by sudden bursts of movement. During these jumps, its maximum instantaneous speed reached 190.1 mm s^−1^, demonstrating a rapid and powerful swimming ability.

## 4. Discussion

The increased availability of high-resolution movement data has significantly advanced the study of animal movement behavior, leading to the development of numerous sophisticated analytical methods [4,10,33]. These advancements have provided researchers with deeper insights into the complex movement patterns of various organisms, including small aquatic species. In this study, we successfully captured the details of the fine-scale movement of the swimming behavior of an adult female copepod *E. japonicus* using a cost-effective high-resolution video recording setup featuring a smartphone under controlled conditions. Previous studies have utilized high-end cameras and expensive commercial software [4,7,20], which often require significantly larger budgets, making them less accessible to researchers with financial constraints. Although the swimming trajectories were successfully extracted using the open-source Fiji software, an advanced image processing platform widely used in biological studies [34], it had not previously been applied to analyses of copepod movement. We developed a video setup that, combined with Fiji’s tracking capabilities, effectively captured the movement trajectories of the copepod, demonstrating its potential for studying small, fast-moving animals. Our method demonstrates that high-quality movement data can be collected using an affordable and accessible approach, making behavioral studies more widely available.

Copepods are known to exhibit two distinct swimming behaviors: cruising, driven by the rhythmic beating of their cephalic feeding appendages, and brief jumps, powered by rapid strokes of their four or five pairs of thoracal swimming legs [35]. In this study, we successfully detected both cruising and jump swimming patterns in *E. japonicus*, with a swimming speed distribution consistent with the findings from our previous study [20]. In a tracking analysis of its movement trajectory in 13 s, we measured a maximum instantaneous swimming speed of 9.0 mm s^−1^ for the female *E. japonicus*, while its cruising swimming speed was predominantly <3 mm s^−1^ [20]. The estimated maximum swimming speed of adult *E. japonicus*, approximately 190 body lengths s^−1^, was similar to that of other small copepod species. For example, the swimming speed of the cyclopoid copepod *Oithona davisae* reaches up to >100 body lengths s^−1^ [10]. The cruising swimming speed of the calanoid copepod *Temora longicornis* was 3.4 mm s^−1^, while its maximum speeds during jump swimming ranged between 25.3 and 107.9 mm s^−1^ [36]. Spontaneous jumps of the calanoid copepod *Acartia tonsa* revealed a maximum swimming speed of approximately 120 mm s^−1^ [37]. According to the length–speed equation for copepod swimming [35], the maximum swimming speed of *E. japonicus* measured in this study is within a similar range (up to 400 body lengths s^−1^) to that for a 1 mm sized copepod. These results validate the reliability of the affordable movement tracking method used in this study, offering an alternative for outreach applications. The consistency of these findings with the established research demonstrates that the tracking system developed here can accurately capture and analyze the fine-scale swimming behaviors of small aquatic organisms. The efficacy of this video tracking setup in accurately capturing the movement trajectories of small, fast-swimming zooplankton suggests its promising potential for broader applications in tracking the movements of various larger animals, such as shrimp, fish, insects, and rodents. This capability could significantly enhance our understanding of complex behaviors, including escape responses, grazing patterns, and predator–prey dynamics. This cost-effective and versatile method holds significant promise for expanding the scope of behavioral studies in various species, thereby advancing our understanding of their movement dynamics in diverse environmental contexts.

In our previous study, the swimming behavior of *E. japonicus* was measured under dark conditions, with illumination provided by infrared-light-emitting diodes (emitting at 870 nm) to avoid the effects of phototropism [20]. Phototropism in freshwater copepods occurs only in response to light flashes or beams, as described above. The lack of a startled response to flashes of light in freshwater copepods is related to the absence of bioluminescence in freshwater environments [29]. To reduce the budget for the video setup, we used uniform visible-light-emitting diodes (emitting at 400–700 nm) to illuminate *E. japonicus* to track its movement trajectories. In this study, *E. japonicus* exhibited swimming behavior similar to that observed in experiments using infrared-light-emitting diodes, under consistent illumination in both cases. These results suggest that consistent, uniform illumination conditions may be critical to preventing phototactic responses in this copepod. Furthermore, the water volume and/or individual density may potentially influence the accuracy of swimming behavior measurements in aquatic animals, as a minimum amount of free swimming space is required. A previous study on the swimming behavior of the small copepod *Eurytemora affinis* in varying water volumes with individual densities ranging from 12 to 320 ind. L^−1^ showed that swimming behavior was independent of the tested density [27]. In this study, a density of *E. japonicus* equivalent to 100 ind. L^−1^ was sufficient to determine swimming behavior. However, a crowding effect on copepods may occur when the density exceeds 500 ind. L^−1^ [38].

Manual video analyses for tracking animal movement, though labor-intensive due to the need for frame-by-frame observations, provide exceptional reliability and accuracy in data analyses [39]. This method ensures that movement trajectories are captured precisely, making it particularly valuable when automated tracking systems struggle to distinguish subtle behaviors or complex patterns. Optical distortion may occur in a video observation system [40]. It is crucial to adjust the camera to ensure that the animal is in focus without optical distortion. Although manual tracking allows for further calibration of the optical distortion, using an assembly with specific lenses may help minimize the optical distortion [41]. Moreover, manual tracking is versatile and can be effectively applied in diverse settings, such as field studies or environments with mixed populations, provided that the target animals can be consistently captured and identified in the video footage. However, its primary limitation lies in its time-consuming nature, as it requires substantial human effort and expertise, potentially restricting its scalability for large datasets or real-time applications. Recently, acoustic telemetry has emerged as a rapidly advancing method for continuously tracking the movement of aquatic animals, such as shrimp, fish, and crabs [42,43]. However, it is unsuitable for tracking small organisms, such as zooplankton. We employed a two-dimensional video analysis system to demonstrate the feasibility of tracking zooplankton’s swimming trajectories. However, this approach has limitations, as it cannot capture complex three-dimensional motion. To address this, developing a cost-effective three-dimensional video analysis system is essential for examining intricate behaviors, such as mating [20], foraging strategies [44], and crowding effects [45].

## 5. Conclusions

In summary, we successfully extracted the swimming trajectory of the fast-swimming mesozooplankton *E. japonicus* using a cost-effective and accessible video setup. This setup provides a practical and affordable solution for studying the movement of small aquatic animals. Moreover, this methodology is not limited to small planktonic organisms: it could also be applied to tracking the movement of larger animals with appropriate modifications. By enabling precise analyses of movement, this approach may contribute to a better understanding of various ecological behaviors.

## Data Availability

The data will be made available on request.

## Supplementary Materials

The following supporting information can be downloaded at https://www.mdpi.com/article/10.3390/mps8040067/s1. Figure S1: A side view of the video setup used to track the swimming behavior of the copepod *Eodiaptomus japonicus*. Video S1: Original video of a swimming *Eodiaptomus japonicus* adult female specimen. Video S2: The extracted trajectory (blue line) of the swimming adult female *Eodiaptomus japonicus*.
