# Supplementary material for: A Simplified Method for Extracting the Movement Trajectories of Small Aquatic Animals
Source: Methods Protoc. 2025 Jun 20;8(4):67. doi: 10.3390/mps8040067 (PMC12388259; doi:10.3390/mps8040067)
Supplement: Supplementary file 1 [file mps-08-00067-s001.zip › Supplementary Materials.pdf]

Supplementary Materials

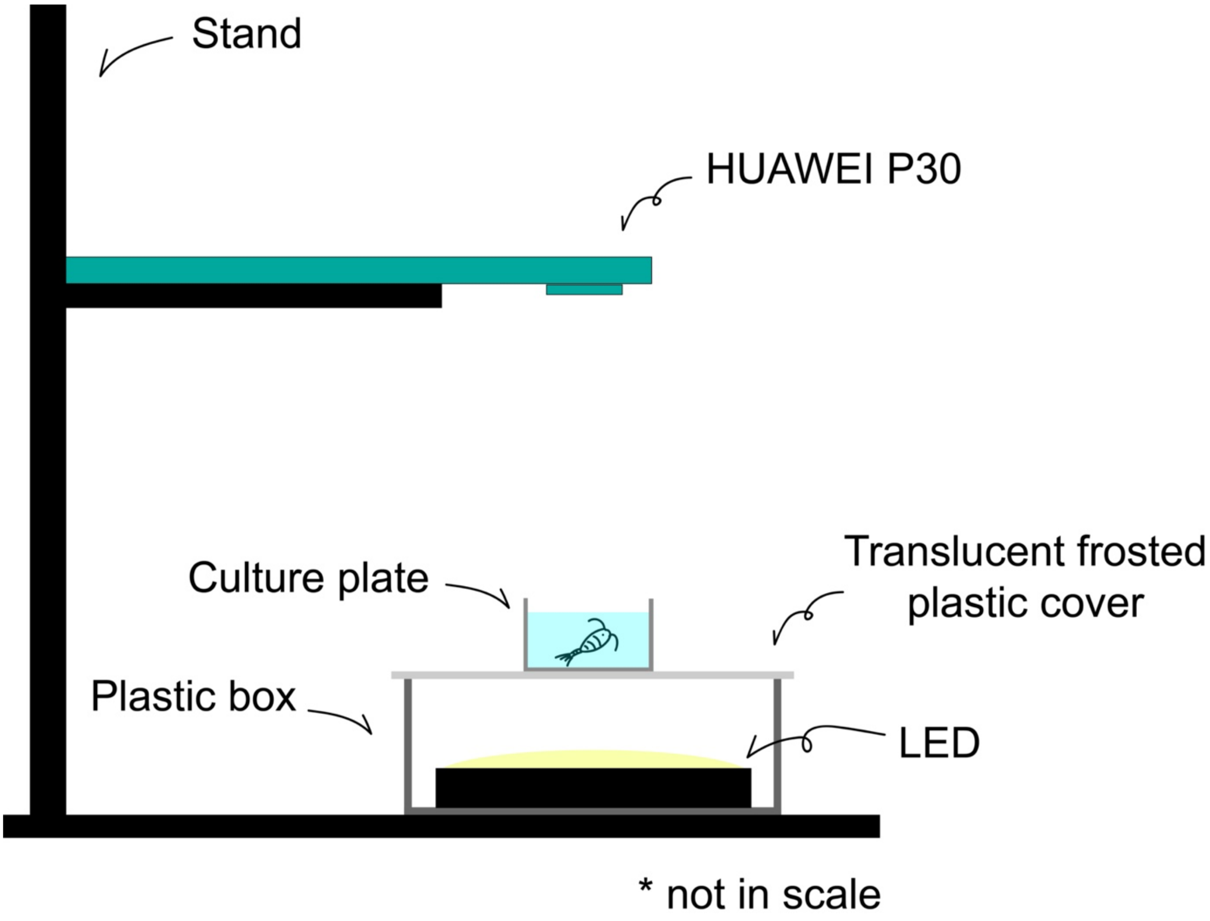

Fig. S1. Side view of the video setup used for tracking the swimming behavior of copepod *Eodiaptomus japonicus*.
